# Economic evaluation of variable rate nitrogen management of canola for zones based on historical yield maps and soil test recommendations

**DOI:** 10.1038/s41598-021-83917-3

**Published:** 2021-02-24

**Authors:** Mohammad Khakbazan, Alan Moulin, Jianzhong Huang

**Affiliations:** Agriculture and Agri-Food Canada (AAFC), Brandon Research and Development Centre, R.R. #3, PO Box 1000a, Brandon, MB R7A 5Y3 Canada

**Keywords:** Plant sciences, Environmental sciences

## Abstract

Canola (*Brassica napus* L.) is a highly valuable crop for Canada’s economy, making the efficient management of canola a priority. A field-scale study was conducted at ten sites between 2014 and 2016 to evaluate the viability of site specific nitrogen (N) management zones (MZ) based on analysis of historical yield maps and soil test recommendations to improve canola productivity and profitability in western Canada. Treatments included factorial combinations of three canola yield zones (low, average, high) by four N rates, replicated four times at each site. The canola yield function had a quadratic form in each field but the effects of MZ varied between fields with positive effects in only a few fields. When ten site-years data were combined, MZ had positive effects on canola performance. On average, MZ of N fertilizer over ten fields generated between $28 to $65 ha^−1^ more net revenue (NR) relative to average yield management. Site-years, which reflect farm management and other farm characteristics had significant effects on yield and NR ranging from − $91 to $352 ha^−1^ compared to a baseline. Nitrogen application under MZs was only reduced by 8% compared to uniform rates. The potential for MZ does exist; however, its effectiveness is highly variable.

## Introduction

Canola (*Brassica napus* L.) is a highly valuable crop for Canada’s economy. The Canadian canola industry was estimated at an annual average value of $15.4 billion between 2007–08 and 2009–10^1^; since then the total economic value of canola to Canada has almost doubled to $26.7 billion per year between 2012 to 2015^2^. Determining methods to more efficiently manage canola is thus very relevant for Canadian producers. The effective application of nitrogen (N) fertilizer through management zones (hereinafter abbreviated MZ or called yield zone) is of particular interest. Nitrogen is the most common limiting nutrient other than water for canola production^3^, and emissions from the production, transportation, storage, delivery, and application of N fertilizer account for approximately 74% of total greenhouse gas (GHG) emissions associated with oilseed crop production^4^. Precision agriculture strategies such as MZ thus have the potential to increase crop yield, improve profitability and nitrogen use efficiency (NUE), and reduce environmental impacts.

Crop yield in the Canadian Prairies, may vary considerably within and between fields due to the spatial distribution of soil fertility^5^, soil electrical conductivity^6–8^, soil moisture^9–11^, and other properties. These variables are often related to landform which directly influences soil properties. Different methods (e.g. remote sensing vs. soil sampling) can affect the viability of precision agriculture^7,12–14^. However, appropriate management can be determined by delineating low and high yield areas based on analysis of yield maps from previous years. Management zones that have been delineated based on combined analysis of crop yield and landform and soil properties, may improve crop yield and economic return by matching fertilizer and other inputs to yield goals for these areas.

Lowenberg-DeBoer^15^ has pointed out that it can be difficult to interpret published results on precision agriculture profitability due to differences in experimental design and assumptions about included costs between different studies. Nevertheless, in an overview of 108 studies on the profitability of precision agriculture, Lambert and Lowenberg-DeBoer^16^ found that 63% of studies indicated positive net returns from precision agriculture, 26% of studies had mixed results, and 11% of studies indicated negative net returns.

Historical yield maps, combined with soil test recommendations are commonly available to producers with GPS equipped yield monitors; therefore, they can be used to delineate MZs within a field. Analysis of the relationship of canola yield to topography and soil properties such as pH, soil organic carbon, or soil conductivity is a lengthy topic, and will be discussed in subsequent publications, outside the scope of this study. Nor was the intent of this study to identify the optimal number of management zones for each field, three zones were determined for all fields.

The research hypothesis of this study was that interaction of N fertilizer rates and MZs significantly increases canola yield with different response curves in each of the management zones. Therefore, delineating a field to different MZs based on analysis of historical yield maps from previous years and soil test recommendations will increase canola yield, economic returns, and NUE. Although there have been many studies on the viability of precision agriculture for a variety of crops, most of these studies have taken place in the United States and elsewhere. Relatively few field-scale studies to assess the effectiveness of N fertilizer through management zones for Canadian canola production have been conducted. Additionally, few studies have combined MZ with temporal trends to investigate profitability at the farm scale. The objective of this research was to assess the economics of yield response for canola due to site specific management of N fertilizer in western Canada based on analysis of historical yield maps, combined with soil test recommendations.

## Results and discussion

### Canola cost of production

Total average annual production costs over the ten sites were $898, $956, and $992 year^−1^ ha^−1^ for low, average, and high zones, respectively (Table 1). The cost differences among zones were due mainly to differences in fertilizer application rates and machinery or transportation costs related to the yield dependent operations. For example, the high zone had higher crop yield and, therefore, higher yield dependent machinery and transportation costs. Fertilizer costs were higher in high zone due to higher application of N rates compared to the average or low zones (Table 1). Operating costs were 66.1, 68, and 69.35% of the total costs for low, average, and high ones, respectively. Fertilizer costs were 16.3, 20.8, and 23.3% of total costs for low, average, and high zones, respectively. Overall, mean fertilizer costs, which made up 20.2% of total cost, were in line with fertilizer costs reported for crop production in western Canada. N fertilizers can comprise more than 31% of the operating costs or 21% of total costs of traditional production systems in western Canada^17^.

**Table 1:** Unit price for inputs and canola product and average costs for canola management zones of ten sites in Manitoba, Saskatchewan and Alberta from 2014 to 2016.

Management zone	N rate	Seed	Machinery	Fertilizer	Pesticide	Operating cost	Total cost
	% of recommended N ha^−1^	$ ha^−1^					
Low	0	155	229	87	78	530	833
Low	50	155	230	128	78	574	877
Low	100	155	232	165	78	615	919
Low	150	155	232	204	78	657	961
Mean of low zone		155	231	146	78	594	898
% of total cost		17.2	25.7	16.3	8.7	66.2	–
Average	0	155	230	87	78	532	835
Average	50	155	232	162	78	613	918
Average	100	155	234	235	78	691	997
Average	150	155	233	310	78	768	1073
Mean of average zone		155	232	199	78	651	956
% of average zone total cost		16.2	24.3	20.8	8.1	68.1	–
High	0	155	232	88	78	535	840
High	50	155	234	185	78	640	946
High	100	155	236	277	78	737	1043
High	150	155	234	373	78	835	1140
Mean of high zone		155	234	231	78	687	992
% of high zone total cost		15.6	23.6	23.3	7.8	69.2	–
Mean of 3 zones		155	232	192	78	644	949
% of total cost		16.3	24.5	20.2	8.2	67.9	–

Canola product and seed		Pesticide ($ kg^−1^ or L^−1^)					
Product ($ kg^−1^)	0.485	Ares	81.63	Liberty	7.88		
Seed ($ kg^−1^)	26.46	Assure	20.40	Matador	179.15		
**Fertilizer ($ kg**^**−1**^**)**		Centurion	15.42	Pardner	27.00		
N	1.24	Exempla	67.71	Proline	139.06		
P_2_O_5_	1.20	Headline	92.31	Roundup	7.92		
K_2_O	0.77	Heat	40.73	Silencer	179.15		
S	0.92	Lance	127.96				

Grain truck	Capacity	1st 5 km	Additional cost after 5 km	Transportation			
	t	$ t^−1^	$ t^−1^ km^−1^				
Truck 1	9.5	9.5	0.92	From field to yard			
Truck 2	13.0	$2.20 t^−1^ km^−1^ from yard to first point of delivery					

### Canola yield, 27 sites 2014 to 2017

**Figure 1:**
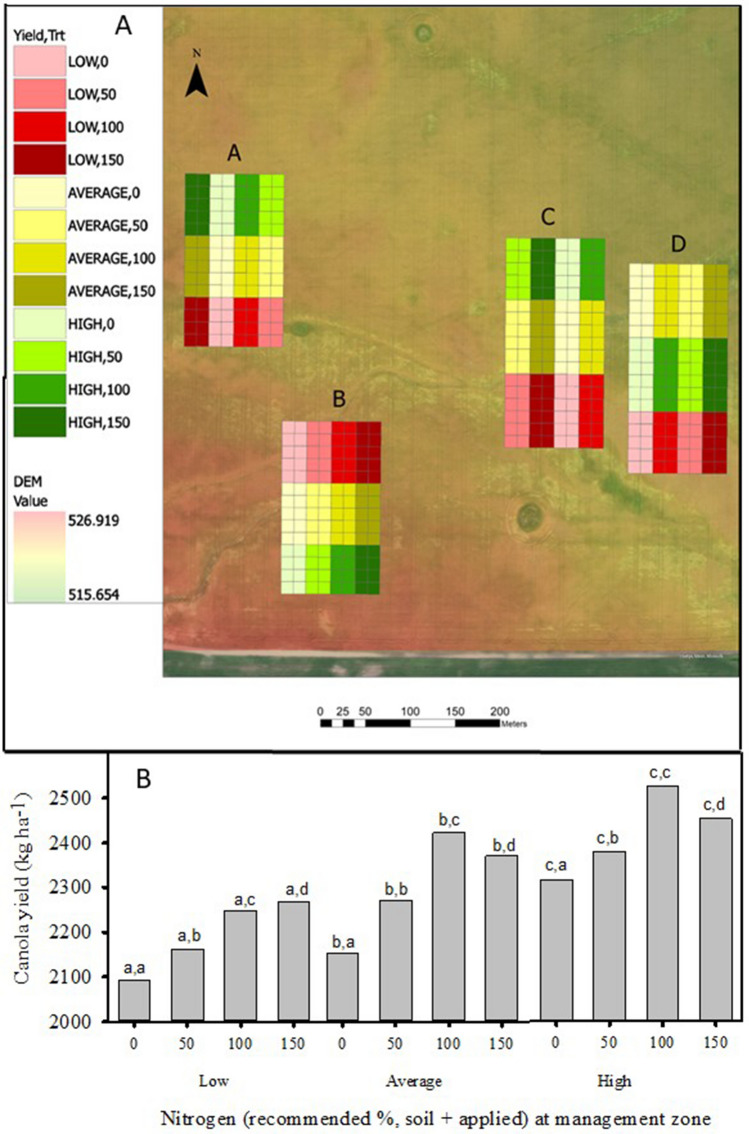
(**A**) Experimental design of yield zones and fertilizer treatments projected over a Digital Elevation Model (m). (**B**) Response of canola seed production to nitrogen fertilizer treatments in three yield zones for analysis of 27 sites in Alberta, Saskatchewan and Manitoba from 2014 to 2017. Multiple pairwise comparisons (Tukey HSD), first letter between fertilizer treatments within zone, second letter for controls between zones [(**A**) ESRI ArcMAP version 10.41, Esri.com. (**B**) SigmaPlot, vision 14, https://systatsoftware.com].

Canola yield was affected by an interaction between fertilizer treatments, management zones and farm site, in analyses of 27 farm fields studied from 2014 to 2017 (Table 2). Yield response to management of N fertilizer was affected by management zones, and field as influenced by covariates such as soil properties, topography, agronomic management, local precipitation and temperature at each site-year. Canola yield within zones increased from the control to 50 and 100% of recommended fertilizer rates in combined analysis across all 27 sites (Fig. 1B). The yield response to N fertilizer was nonlinear, in the combined analysis for all fields, canola yield did not increase from 100 to 150% of the recommended fertilizer rates within the average and high zones (Fig. 1B). Canola yield was higher in the high zone compared to the low yield zones, with intermediate values for average zones.

**Table 2:** Linear model analysis (maximum likelihood mixed model) of canola yield for 27 site-years from 2014 to 2017 and canola yield and net revenue (NR) for ten site-year from 2014 to 2016 as affected by field site, management zones, and nitrogen fertilizer in Manitoba, Saskatchewan and Alberta, Canada.

Source	Canola yield for 27 site-year from 2014 to 2017					
	df	F ratio	Prob > F			
Zone	2	96.2	< 0.0001			
Fertilizer	3	243.9	< 0.0001			
Field	26	438.0	< 0.0001			
Fertilizer by zone	6	3.6	0.0013			
Zone by field	52	11.9	< 0.0001			
Fertilizer by field	78	18.3	< 0.0001			
Fertilizer by zone by field	156	3.1	< 0.0001			

Year	Canola yield and NR for 10 site-year from 2014 to 2016					
	Field site		Management zone		Fertilizer treatment	
	df	p value	df	p value	df	p value
**Yield**						
2014	2	< 0.0001	2	< 0.0001	3	< 0.0001
2015	2	< 0.0001	2	0.0295	3	0.0003
2016	3	< 0.0001	2	< 0.0001	3	< 0.0001
2014–2016	8	< 0.0001	2	< 0.0001	3	< 0.0001
**NR**						
2014	2	< 0.0001	2	0.005	3	0.1367
2015	2	< 0.0001	2	0.2681	3	0.0021
2016	3	< 0.0001	2	0.0032	3	< 0.0001
2014–2016	8	< 0.0001	2	0.0168	3	0.0017

### Canola yield and NR by sites in 2014

**Figure 2:**
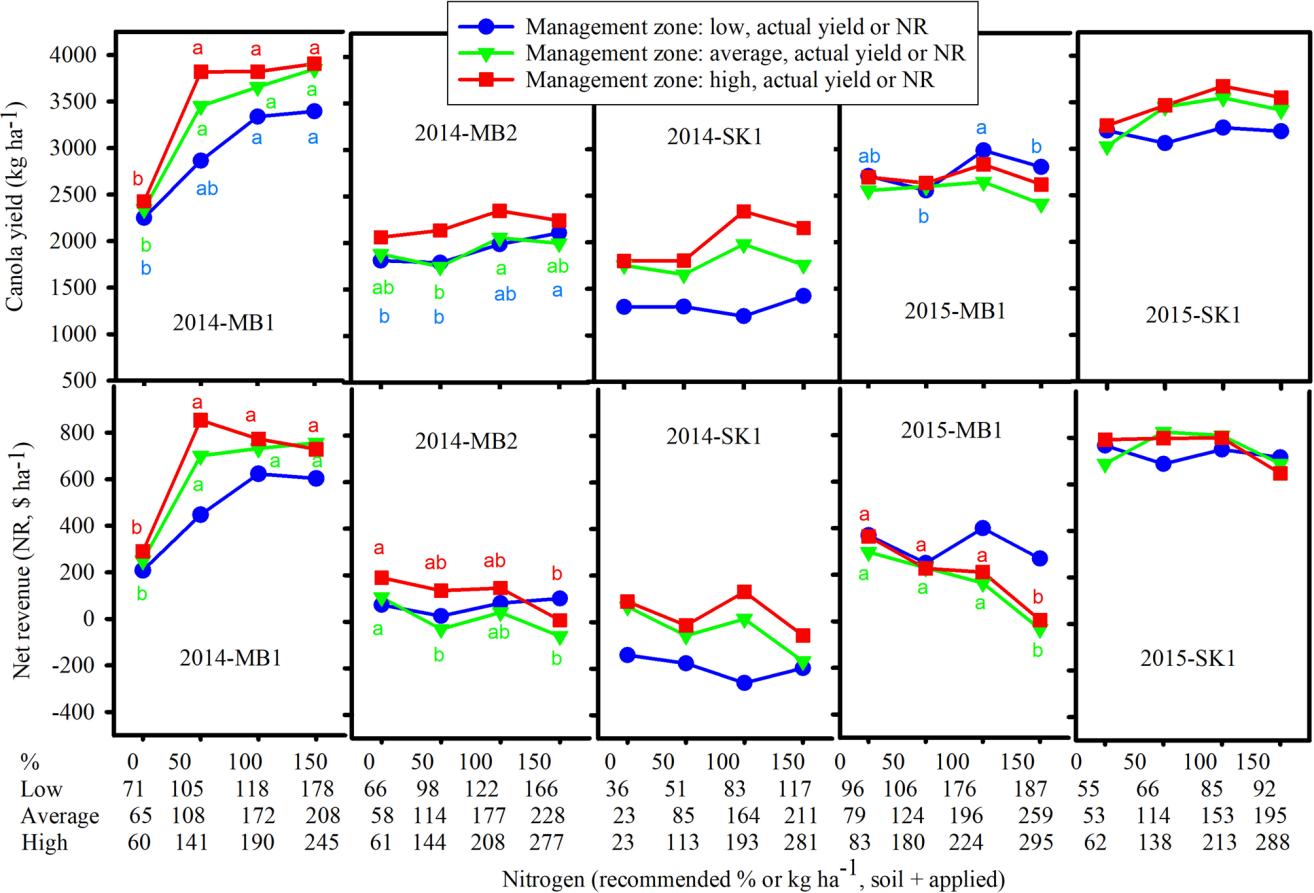
Response of canola yield and net revenue to nitrogen fertilizer treatments in three canola management zones at 2014-MB1, 2014-MB2, 2014-SK1, 2015-MB1 and 2015-SK1. Means followed by the same letters for each canola management zone are not significant (*p* > 0.05) (SigmaPlot, vision 14, https://systatsoftware.com).

Canola yield and NR within zones in 2014 were affected by recommended N in Manitoba but not in Saskatchewan fields (Fig. 2). In all zones of 2014-MB1, yield and NR of canola were greater for the 100% recommended N treatment compared to 0% N (Fig. 2). In high and average yielding zones of 2014-MB2 and 2014-SK1, NR was maximized at 0% N and more Ns did not generate additional NR. In the low zone, N for maximum canola yield (and N for maximum NR) were 118 (118), 166 (66), and 36 (26) kg N ha^−1^ for 2014-MB1, 2014-MB2, and 2014-SK1, respectively. In the average yield zone, N for maximum canola yield (and N for maximum NR) were 208 (172), 177 (58), and 164 (23) kg N ha^−1^ for 2014-MB1, 2014-MB2, and 2014-SK1, respectively. In the high yield zone, N for maximum canola yield (and N for maximum NR) were 141 (141), 208 (61), and 193 (23) kg N ha^−1^ for 2014-MB1, 2014-MB2, and 2014-SK1, respectively. These Ns included both soil and N application. The Ns to achieve maximum NR were less than Ns for maximum yield.

Within 50–100% recommended N rates, the significant effect of management zone on canola yield and NR was found only for 2014-SK1 (Fig. 4). Yields in high and average zones (2068 and 1817 kg ha^−1^, respectively) were higher than yield in low zone (1258 kg ha^−1^) for the 2014-SK1. Similarly, NRs in high and average zones ($58 and − $22 ha^−1^, respectively) were higher than NR in low zone (− $219 ha^−1^).

The linear and quadratic response of predicted yield and NR to N rate (soil plus N applied) for 2014-MB1, 2014-MB2 and 2014-SK1 was regressed with average zone as a baseline (Table 3). Linear and quadratic terms for N were both significant for 2014-MB1. However, coefficients for high zone (difference between high zone and average zone) and low zone (difference between low zone and average zone) were not significant. At 2014-MB2, the effect of N fertilizer on yield was linearly significant along with effect of high zone on both yield (253 kg ha^−1^) and NR ($128 ha^−1^). At 2014-SK1, the regression coefficient for low zone was significant on both yield (− 408 kg ha^−1^) and NR (− $185 ha^−1^).

**Table 3:** Regression for canola yield and net revenue (NR) for each site, for pooled each year, and for combined three years (2014–2016) of ten sites data in Manitoba (MB), Saskatchewan (SK) and Alberta (AB), Canada. Each site includes low, average, and high canola management zones.

Variable	Field experimental site										
	2014			2015			2016				
	MB1	MB2	SK1	MB1	SK1	SK2	MB1	MB2	MB3	AB1	
**Yield (kg ha**^**−1**^**)**											
N	24.19*	5.87*	1.25	7.27*	6.65*	14.62*	6.76	6.65*	10.66*	2.25	
N^2^	− 0.054*	− 0.012	0.001	− 0.019*	− 0.015**	− 0.04*	− 0.021	− 0.015*	− 0.023*	− 0.008	
Low zone	− 248	58	− 408*	199	− 34	67	− 190	− 302*	− 82	− 30	
High zone	66	253*	190	173	91	109	164	287*	87	63	
Constant	1209*	1379*	1627*	1978*	2795*	1656*	2059*	901*	865*	2694*	
**NR ($ ha**^**−1**^**)**											
N	10.34*	1.6	− 0.64	2.07	1.95	5.4*	2.17	1.68**	3.72*	− 0.7	
N^2^	− 0.026*	− 0.006	0.0003	− 0.009**	− 0.007**	− 0.018*	− 0.011	− 0.008*	− 0.011*	− 0.004	
Low zone	− 111	46	− 185*	124**	− 16	33	− 89	− 160*	− 41	− 11	
High zone	29	128*	91	87	50	39	87	165*	21	32	
Constant	− 228	− 75	35	102	644*	141	172	− 349*	− 425*	661*	

2014 (3 sites)			2015 (3 sites)			2016 (4 sites)			2014–2016 (10 sites)		
Variable	Yield	NR	Variable	Yield	NR	Variable	Yield	NR	Variable	Yield	NR
	kg ha^−1^	$ ha^−1^		kg ha^−1^	$ ha^−1^		kg ha^−1^	$ ha^−1^		kg ha^−1^	$ ha^−1^
N	7.54*	2.35*	N	9.42*	3.19*	N	4.98*	0.76	N	7.04*	1.93*
N^2^	− 0.013	− 0.006	N^2^	− 0.024*	− 0.011*	N^2^	− 0.01*	− 0.004*	N^2^	− 0.016*	− 0.007*
Low zone	− 166	− 67	Low zone	71	45	Low zone	− 142*	− 70*	Low zone	− 91*	− 37**
High zone	165	80	High zone	125*	58*	High zone	120*	58*	High zone	135*	65*
MB1	1250*	510*	SK2	− 100*	− 46*	AB1	48*	37*	2014-MB1	751*	352*
SK1	− 58**	− 34*	MB1	− 107*	− 73*	MB2	− 95*	− 47*	2014-MB2	− 246*	− 77*
Constant	1275*	− 114	Constant	2590*	547*	MB3	− 61*	− 34*	2014-SK1	− 230*	− 91*
						Constant	2064*	223*	2015-SK1	177*	106*
									2015-SK2	49.57*	43.47*
									2015-MB1	17.96**	2.662
									2016-AB1	51*	39*
									2016-MB2	− 95*	− 47*
									2016-MB3	− 60*	− 33*
									Constant	1877*	113*

Average canola management zone was selected as base zone for regression analysis for each individual site. When data were pooled together for regression analysis for each year, site 2014-MB2, 2015-SK1 and 2016-MB1 were selected as base site for 2014, 2015 and 2016, respectively. When three years of data were pooled together, site 2016-MB1 was selected as base site for regression analysis. *p ≤ 0.05 and **0.5 < p < 0.1.

### Canola yield and NR by sites in 2015

**Figure 3:**
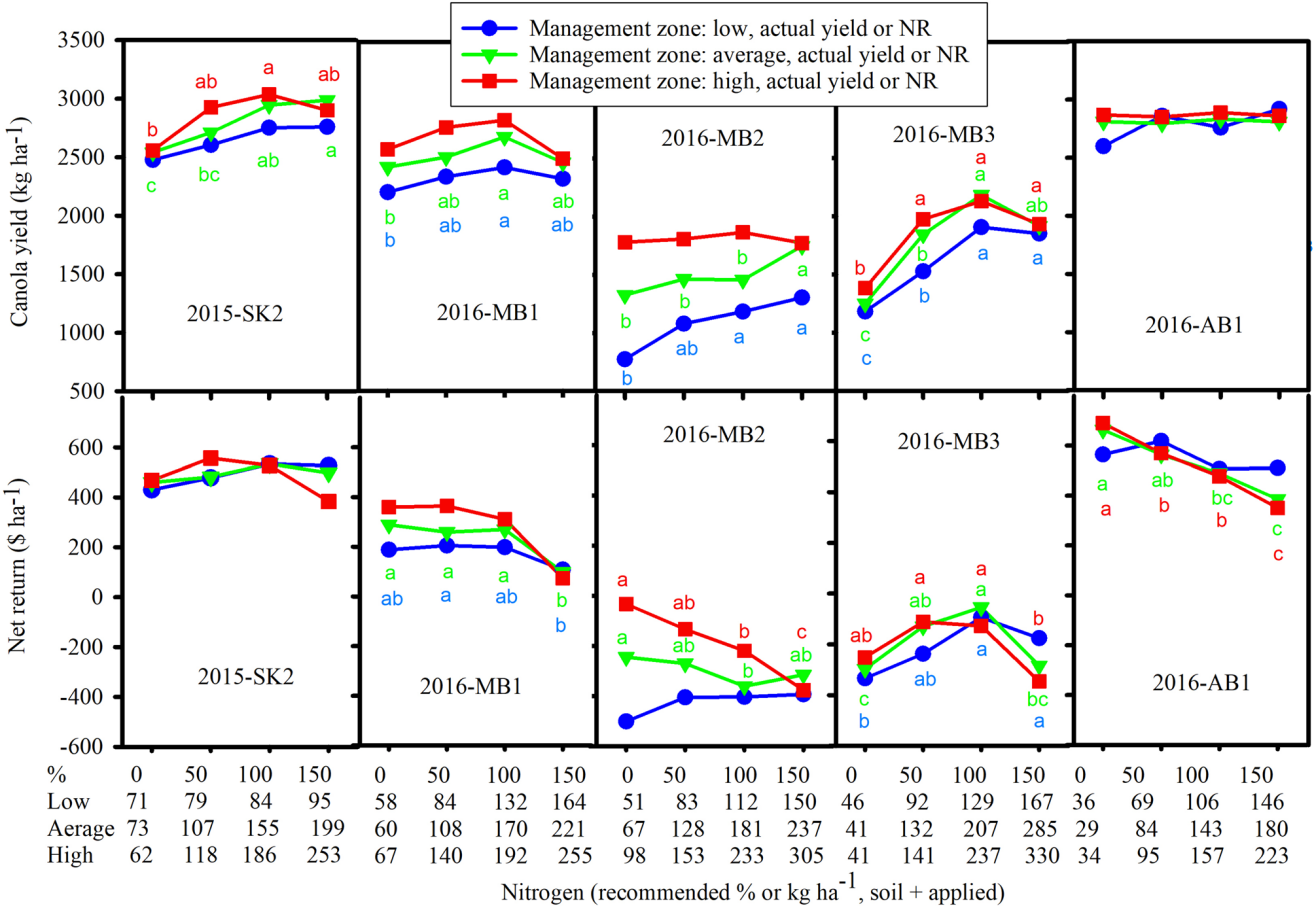
Response of canola yield and net revenue to nitrogen fertilizer treatments in three canola management zones at 2015-SK2, 2016-MB1, 2016-MB2, 2016-MB3 and 2016-AB1. Means followed by the same letters for each canola management zone are not significant (*p* > 0.05) (SigmaPlot, vision 14, https://systatsoftware.com).

ANOVA analysis showed that at low zone, differences in yield between different recommended N rates were only significant at 2015-MB1 but not in 2015-SK1 and 2015-SK2 (Figs. 2 and 3). In the average and high zones of 2015-SK2, a higher canola yield at 100% recommended N compared to 0% N was statistically significant. In the low yield zone, canola yield was maximized at 176, 55, and 84 kg N ha^−1^ for 2015-MB1, 2015-SK1, and 2015-SK2, respectively. In the average yield zone, canola yield was maximized at 196, 153, and 155 kg N ha^−1^ while NR was maximized at 79, 114, and 155 kg N ha^−1^ for 2015-MB1, 2015-SK1, and 2015-SK2, respectively. In the high yield zone, canola yield was maximized at 224, 213, and 186 kg N ha^−1^ while NR was maximized at 83, 62, and 118 kg N ha^−1^ for 2015-MB1, 2015-SK1, and 2015-SK2, respectively (Figs. 2 and 3). These Ns include both soil and N application.

**Figure 4:**
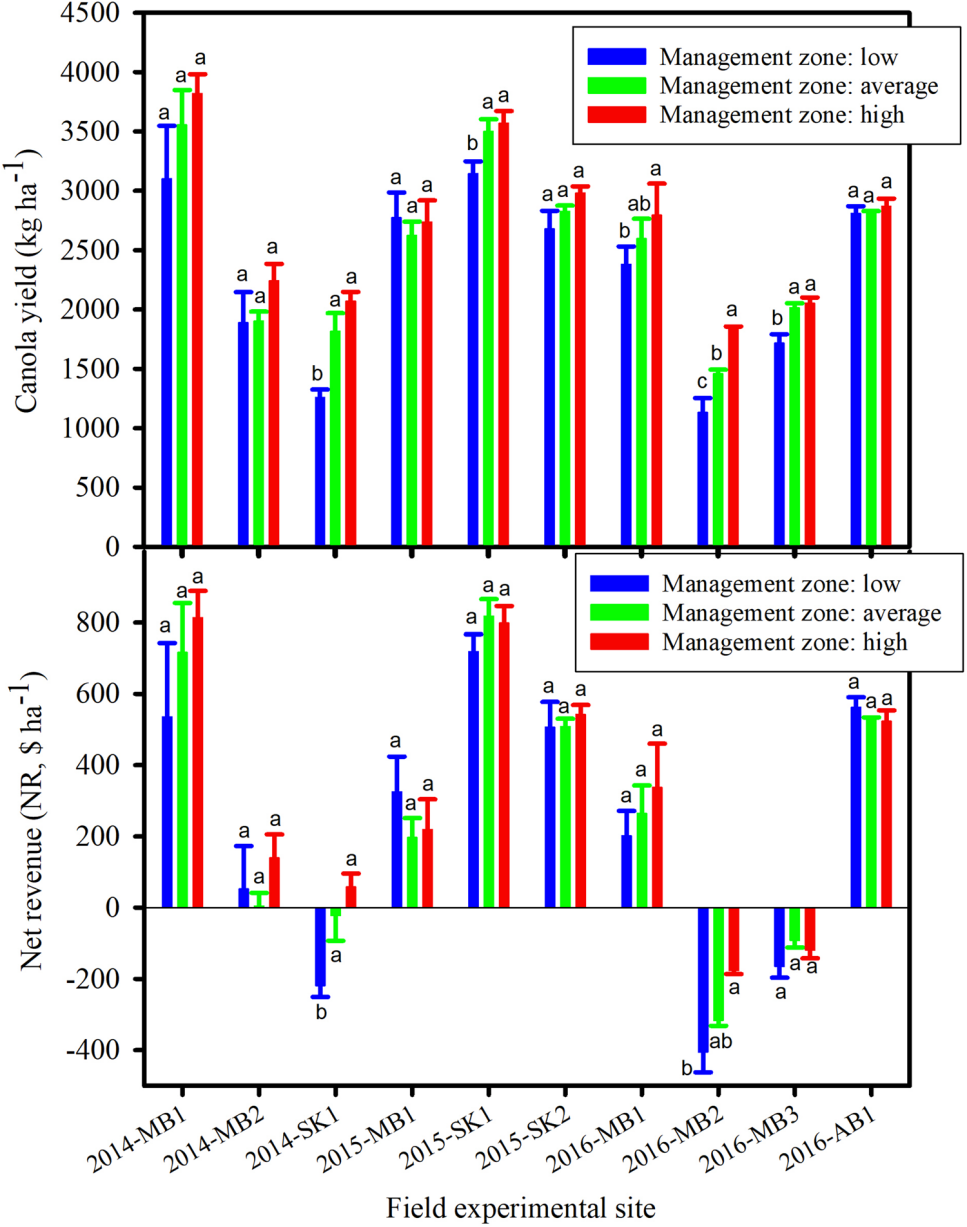
Canola yield and net revenue at 50–100% recommended nitrogen fertilizer treatments in three canola management zones for ten field experimental sites from 2014 to 2016. The same letters on the top of bars for each site are not significant among management zones (*p* > 0.05) (SigmaPlot, vision 14, https://systatsoftware.com).

The significant effect of management zone on canola yield was found for 50–100% N rates only at 2015-SK1 where yield in low zone (3144 kg ha^−1^) was lower than yields in high and average zones (3570 and 3500 kg ha^−1^, respectively) (Fig. 4).

The regression coefficients and significance tests are listed in Table 3. The effects of zones on canola yield were not significant; however, low zone at 2015-MB1 provided higher NR compared to average zone because more soil N was available at 0% N.

### Canola yield and NR by sites in 2016

Statistical analysis showed that in the low zone of Manitoba sites in 2016, increasing N to 100% N increased canola yield compared to 0% N; however, increasing N rates did not increase NRs except at 2016-MB3 where increasing N to 100% recommended N generated more NR than 0% N. In the average zone, increasing N from 0 to 100% N increased canola yield at 2016-MB1 and at 2016-MB3 but not at 2016-MB2. Regardless of the locations, increasing N did not increase NR in the average zones of 2016 sites except at 2016-MB3. In all four sites in 2016, application of more N in high zones did not increase NRs, perhaps due in part to higher soil available N in these sites (Fig. 3). Canola yield in the high zone of 2016-MB3 was higher for the 50 to 150% rate of N compared to 0% N; however, the increase yield was not enough to cover the extra cost of N fertilizer. In the low yield zone, canola yield was maximized at 132, 150, 129, and 69 kg N ha^−1^ while NR was maximized at 58, 83, 129, and 69 kg N ha^−1^ for 2016-MB1, 2016-MB2, 2016-MB3, and 2016-AB1, respectively. In the average yield zone, canola yield was maximized at 170, 237, 207, and 29 kg N ha^−1^ while NR was maximized at 60, 67, 207, and 29 kg N ha^−1^ for 2016-MB1, 2016-MB2, 2016-MB3, and 2016-AB1, respectively. In the high yield zone, canola yield was maximized at 192, 98, 237, and 34 kg N ha^−1^ while NR was maximized at 67, 98, 141, and 34 kg N ha^−1^ for 2016-MB1, 2016-MB2, 2016-MB3, and 2016-AB1, respectively.

The significant effect of management zone on canola yield and NR was found for 50–100% N rates (Fig. 4). At 2016-MB1, canola yield was higher at the high zone (2796 kg ha^−1^) compared to the low zone (2381 kg ha^−1^). At 2016-MB2, canola yield was higher at high zone (1837 kg ha^−1^) compared to low and average zones (1132 and 1460 kg ha^−1^, respectively), and yield at the average zone was higher than yield at the low zone (Fig. 4). At 2016-MB2, NR was higher at the high zone (− $177 ha^−1^) than the low zone (− $405 ha^−1^). At 2016-MB3, yields at the high and average zones (2053 and 2014 kg ha^−1^, respectively) were both higher than yield at the low zones (1717 kg ha^−1^) (Fig. 4).

At 2016-MB2, linear and quadratic coefficients for N, high zone (287 kg ha^−1^), and low zone (− 302 kg ha^−1^) were significant on yield (Table 4). At this location, only quadratic coefficients of N, high zone ($165 ha^−1^), and low zone (− $160 ha^−1^) were significant for NR. At 2016-MB3, linear and quadratic coefficients for N were also significant.

**Table 4:** Nitrogen (N) at 100% recommended N application and total N rate (soil plus applied) at maximum yield and maximum net revenue for canola production at low, average, and high canola management zones in Manitoba (MB), Saskatchewan (SK) and Alberta (AB), Canada from 2014 to 2016 as well as Manitoba agricultural services corporation (MASC) average applied N at risk area five in Manitoba.

N	N application management					N (kg ha^−1^)							
		2014				2015			2016				2014–2016
		MB1		MB2	SK1	MB1	SK1	SK2	AB1	MB1	MB2	MB3	Mean
Applied N at 100% recommended	Uniform	103		134	129	134	123	118	101	116	136	178	126
	MZ	103		123	119	127	118	107	98	110	128	164	119
	Difference	0		11	10	7	5	11	3	6	8	14	7
N (soil + applied) at maximum yield	Uniform	208		177	164	196	153	155	29	170	237	207	170
	MZ	156		184	131	199	140	142	44	165	162	191	151
	Difference	52		− 7	33	− 3	13	13	− 15	5	75	16	19
N (soil + applied) at maximum net revenue	Uniform	172		58	23	79	114	155	29	60	67	207	96
	MZ	144		62	24	113	77	119	44	62	83	159	89
	Difference	28		− 4	− 1	− 34	37	36	− 15	− 2	− 16	48	7
MASC average applied N at Risk Area five in Manitoba				117						118			

*Uniform *uniform N rate at average canola management zones, *MZ *average N rate at low, average, and high canola management zones, *MASC* Manitoba Agricultural Services Corporation^17^.

### Canola yield and NR by sites in 2014, 2015, and 2016

Overall, regression results showed that zones were not significant at 2014-MB1, but the high zone in 2014-MB2 produced higher canola yield (253 kg ha^−1^) and higher NR ($128 ha^−1^) than the average zone. The low zone in 2014-SK1 produced less canola yield (408 kg ha^−1^) and less NR ($185 ha^−1^) compared to the average zone (Table 3). Results for sites in 2015 showed that zones were not significant except for the effect of low zone on NR in 2015-MB1 (Table 3). Similarly, zones were not significant in 2016 except in 2016-MB2 where high zone produced more yield (287 kg ha^−1^) and NR ($165 ha^−1^) and low zone less yield (302 kg ha^−1^) and less NR ($160 ha^−1^) than the average zone (Table 3). Results also showed that Ns to achieve maximum NR were less than Ns for maximum yield. Other studies have found similar mixed results. Beckie et al.^18^ found net returns from precision farming to be about $10 ha^−1^ greater compared to uniform rate N fertilization of wheat (*Triticum aestivum* L.), canola, and flax (*Linum usitatissimum* L.) grown in black soil climate near Prince Albert, Saskatchewan, Canada. Similarly, in Inverleigh, Australia, Torpy^19^ found that precision agriculture had a beneficial impact on investment as canola yield differences were higher in paddocks with high spatial variability. A study on the effect of variable rate application on cereal grains in Oklahoma and Virginia found that the increase in revenue from precision agriculture was more than sufficient to cover the technology costs^20^. Conversely, Long et al.^21,22^ found net returns from precision agriculture of hard red spring wheat in northern Montana to be reduced by up to $27.97 ha^−1^ compared to net returns under uniform N management. Boyer et al.^23^ found no statistical differences in wheat yield and net return between variable rate N and uniform rate application in Oklahoma. A study in eastern Washington found no significant differences in winter wheat yield between uniform and variable rate N application but found that less N fertilizer was applied under precision application in two out of three management zones^24^. A three-year study in Saskatchewan, Canada found also little economic rationale for precision agriculture due to the unpredictable nature of wheat yield response to varied N rates^25^. Several studies have found that fertilizer cost savings have a greater impact on the economic benefits of precision farming than do increases in grain yield^26–28^.

### Canola yield and NR, annual regression analyses for 2014, 2015, and 2016

The quadratic response of predicted yield to N application for each individual year, assuming fixed effects of zones and location within each year, were regressed (Table 3). In 2014, although the canola yield function was quadratic in form similar to results for yield in combined analyses for 2014–2017 (Fig. 1B), only the linear coefficient and the coefficients for locations were significant. 2014-MB1 produced 1250 kg ha^−1^ higher canola yield than 2014-MB2, while 2014-SK1 produced 58 kg ha^−1^ less canola than 2014-MB2. Canola yield for both 2014-MB1 and 2014-MB2 sites were higher than yield at 2014-SK1 due, in part, to higher growing season precipitations in Manitoba sites. In 2014, growing season precipitation in Manitoba was 17% higher than the growing season precipitation of the same year in Saskatchewan (data not shown). NR for 2014 showed similar patterns with only linear term for N and locations being significant. 2014-MB1 generated $510 ha^−1^ higher canola NR than 2014-MB2, while 2014-SK1 generated $34 ha^−1^ less canola NR than 2014-MB2. In 2015, coefficients for linear and quadratic effects of nitrogen and high zone on canola yield and NR and effects of locations were significant but not the effects of low zone. In 2016, both yield and NR functions were quadratic in form and the effects of all coefficients of independent variable on canola yield and NR were significant except the linear effect of N on NR. The high zone produced 120 kg ha^−1^ higher canola yield or $58 ha^−1^ higher canola NR than the average zone. Conversely, the low zone produced 142 kg ha^−1^ less canola yield or $70 ha^−1^ less canola NR than the average zone. Growing season precipitation in 2016 in Manitoba sites were 68% higher than the growing season precipitation of the same year in Alberta; however, this did not result in more canola yield in Manitoba (data not shown). In all three years (2014, 2015, 2016), coefficients for sites which captured combination effects of site management and site specifics were significant, reflecting different farm management and specifics and including soil and environment at each site. Overall, regression results showed that the coefficients for zones though all had expected signs, were only significant for high zone in 2015 and for both high and low zones in 2016. This could be explained by variability of fertilizer rates for MZs between fields, by uniform yield for some sites, and by factors such as soil properties.

The ANOVA results for the effects of nitrogen fertilizer, canola management zones, and locations on canola yield and NR were somewhat different than the regression analysis (Table 2). In regression, zones and locations were fixed effects with indicator variables; however, in the ANOVA analysis, no baselines were selected and only the effects of these variables on canola yield or NR were tested. ANOVA results for each individual year showed that the p values for the effects of nitrogen, zones, and locations on canola yield and NR were significant except the effects of nitrogen on NR in 2014 and zones on NR in 2015 were not significant.

### Canola yield and NR, combined analyses for 2014 to 2016

The quadratic response of predicted yield to N application for all site-years was regressed (Table 3). Both the linear and quadratic effects of nitrogen on canola yield were found to be significant. The high zone produced 135 kg ha^−1^ higher, while yield in the low zone was 91 kg ha^−1^ lower than the average zone. 2016-MB1 was assumed as a baseline and other site-years were compared to this baseline. Coefficients for site-years which capture a combination of effects of year and site management, and site specifics were significant. This reflects different farm management, site specifics and soil and environmental aspects at each site. For example, 2016-MB2 produced 95 kg ha^−1^ less canola yield than 2016-MB1, while 2015-SK1 produced 177 kg ha^−1^ more canola than 2016-MB1. Growing season precipitation in 2016 in Manitoba was 14% higher than the 2014 growing season precipitation in Manitoba and 20% higher than the 2015 growing season precipitation in Saskatchewan (data not shown); however, this higher precipitation did not result in more canola yield compared to sites in 2014 and 2015 in Manitoba and Saskatchewan.

For all 3-year (10 sites) combined, the linear and quadratic coefficients for N on canola NR were significant (Table 3). The high zone generated $65 ha^−1^ higher canola NR than the average zone. The effect of the low zone on canola yield and NR as compared to average zone was negative but was significant only at 10% p value. The low zone reduced canola NR by $37 ha^−1^ compared to NR of canola in the average zone. Coefficients for farm sites as compared to 2016-MB1 were significant for all farms, reflecting contribution of different farm management and soil and environmental specifics at each site. For example, 2016-MB2 generated $47 ha^−1^ less canola NR than 2016-MB1, while 2015-SK1 generated $106 ha^−1^ more canola NR than 2016-MB1.

### Nitrogen application under MZ as compared to uniform management

The response of canola yield to variable and uniform N management varied between years and farms (Table 4). For example, in 2014-MB2, 123 kg ha^−1^ N was applied at 100% N treatment over the three zones, but 134 kg ha^−1^ N was applied under a uniform application. Or, in 2016-MB2, 128 kg ha^−1^ N was applied at 100% N treatment over the three zones, but 136 kg ha^−1^ N was applied under a uniform application. As results showed, the N application was reduced on average by only 7 kg N ha^−1^ under MZ. This was based on the actual amount of N applied. If, however, N applied was based on a yield maximizing scenario (Figs. 2 and 3), reduction in N and therefore NUE will be more significant. For a yield maximizing scenario, N was on average 18 kg N ha^−1^ less under management zones compared to uniform application. Studies on the environmental benefits of precision agriculture as compared to conventional fertilizer application found significant benefits with precision agriculture^20,27,29–32^. Holzapfel et al.^30^ reported that sensor-based N management of canola grown in Saskatchewan, Canada increased agronomic NUE 33% of the time. Similarly, Raun et al.^20^ found that precision agriculture increased NUE by over 15% on average for cereal grains grown in Oklahoma and Virginia. Diacono et al.^31^ reported up to 368% increased in NUE for wheat using real-time sensor-based N management systems as compared to conventional farming practices.

## Conclusions

Variable application of N fertilizer based on management zones was not effective in all fields, due to variability between sites. Overall, when ten fields were combined, management zones of N fertilizer increased canola NR by up to $65 ha^−1^ compared to average yield management. Site-year or locations of the fields over 2014–2016 which reflect farm management and other specifics like soil and environment at each site had significant effects on yield and NR when compared to a baseline ranging from − $91 to $352 ha^−1^. Rates of N fertilizer applied under MZ were about 8% less than N applied under uniform rates as nutrient supply matched more effectively with crop demand. We attribute this modest N application between uniform and management zones to similar yield goals for different zones, and spatial distribution of MZs, where fertilizer rates in high zones likely offset low rates in low zones. The potential for spatial management of N fertilizer does exist; though the application of three management zones based on historical yield maps combined with soil test recommendations does not account for variability in all fields and is not consistently effective.

## Materials and methods

### Experimental fields

Research was conducted with field scale producer’s equipment in western Canada (Alberta, Lat. at 52° 26′ 56.006″ N and Long. at 113° 45′ 21.005″ W, Saskatchewan, Lat. at 52° 49′ 00.000″ N and Long. at 104° 36′ 00.000″ W, and Manitoba Lat. at 49° 11′ 15.500″ N and Long. at 98° 05′ 02.200″ W) during 2014, 2015, and 2016. Experimental field, consisting of the factorial combination of three canola yield zones (low, average, and high yield zones) by four N rates (0, 50, 100 and 150% of recommended rates^33^ were replicated four times at each site (hereinafter called site-year or site or location). The ten sites where experimental fields were established included two sites in Manitoba and one site in Saskatchewan in 2014 (hereinafter called 2014-MB1, 2014-MB2, 2014-SK1, respectively), one site in Manitoba and two sites in Saskatchewan in 2015 (hereinafter called 2015-MB1, 2015-SK1, 2015-SK2, respectively), and three sites in Manitoba and one site in Alberta in 2016 (hereinafter called 2016-MB1, 2016-MB2, 2016-MB3, 2016-AB1, respectively). These ten sites were selected, for analysis of yield and net revenue, from a larger agronomic data set of 27 sites in the Canadian Prairies from a research project conducted from 2014 to 2017. This economic subset was selected based on the availability of suitable economic data for the fields, as sufficient cost of suitable production and agronomic management data were not available for the remainder of the sites. Producers set yield goals for each field that were adjusted for each zone based on historical data. Fields were selected from those volunteered by producers based on the availability of 3–5 years of yield maps, variable rate equipment and GPS equipped combines, and logistical constraints such as travel distance for sample collection. Terrain attributes were calculated for each field, and soil properties including pH, conductivity, soil organic carbon were sampled. Meteorological data were obtained from nearby Environment Canada meteorological stations, and weed and plant disease were measured in each field. Canola varieties were identified by most producers in the project. However, detailed spatial analysis of yield, terrain attributes, canola varieties, soil properties such as pH, conductivity and organic carbon, weeds and plant disease and meteorological data will be addressed in subsequent publications, in analyses which are outside the scope of the objectives for economic analysis.

The low, average, and high yield zones were determined for all fields based on analysis of 3–5 years yield maps with K-means cluster spatial analysis of normalized yields in SMS advanced^34^. The cluster analysis separated the fields into zones with low, average and high normalized yield, based on historical data. The number of zones (3) was predetermined prior to cluster analysis, in order to provide sufficient space for plots, replicates and logistics for field-scale seeders and combines. Four nitrogen fertilizer treatments nested within each zone in each of four replicates (A, B, C and D) in each field (Fig. 1A). Nitrogen fertilizer treatments as urea or blends were based on soil tests for N (2 M KCl extract) and producer yield goals for each zone. Fertilizer rates were determined for soil samples collected in the fall or spring prior to seeding canola, from each plot and subplot in each field, and were based on recommendations in Manitoba^33^. These rates were applied in the spring based on the producer’s seeding and production system for all canola varieties. Target yields were identified by producers for the field average based on their experience, and were closely related to those determined from the historical yield maps. Target yields were further adjusted for low and high zones, relative to the average. Phosphorus, potassium (K), and sulphate (S) fertilizer rates were based on soil test P, K and S, were uniformly applied in all treatments, and judged sufficient for the study. Mono-ammonium phosphorus (MAP) was applied based on soil test, to all treatments. The experimental design addresses the yield response and economics of canola for farm fields in response to two fixed factors, fertilizer rates (ordinal) nested within management zones (ordinal) based on soil test analysis, in a strip split plot design in linear models which specify replicates, treatments, fields and interactions. Statistical analyses of canola yield for 2014 to 2017 were conducted with JMP Pro v 15.0.0^35^ based on linear models calculated with maximum likelihood. Yield was geospatially interpolated (Empirical Bayesian Kriging, EBK) for subplots within plots (Geostatistical Analyst, ArcGIS Desktop 10.41, Environmental Systems Research Institute, ESRI) from yield monitor data.

Weeds, disease and insects were managed by the producers with the appropriate pesticides, in field scale applications which included the plots and the rest of the field. Canola hybrid varieties were selected by the producers and varied between fields. Canola varieties were 46H75 (2014-MB1), L130 (2014-MB2), 5440 (2014-SK1), L165H (2015-MB1), 45H31 or PR530 (2015-SK1, 2015-SK2), L252 (2016-AB1), L140P (2016-MB1), L165H (2016-MB2, 2016-MB3). Experiments were conducted by producers at the field scale, canola varieties, equipment, agronomic management and fertilizer used for production varied between farms. All canola production data were collected with GPS equipped yield monitors and were calibrated on an annual basis with commercial scales or weigh wagons. Yield monitor variables, including site accuracy and outliers, and alignment of GPS points along harvest paths were reviewed for quality control. Yield monitor data, with values greater than the 97.5% quantile, and less than the 0.1% quantile, were removed. The remaining points were averaged by plot for statistical analysis of economic return, but retained for regression analysis of yield response to N fertilizer.

### Economic analysis

Net revenue (NR), as calculated by Zentner et al.^36^, was defined as the revenue remaining after paying for all costs. This included variable costs (i.e. seed, nutrients, weed and disease control, transportation, fuel and oil, repairs, miscellaneous expenses, land taxes, interest cost on variable inputs), machinery and buildings land investment and ownership costs (depreciation, interest on investment, insurance and housing), and labour costs. Costs and NR were estimated using 2019 input prices (Table 1). For each site, the NR was averaged and expressed in CND $ ha^−1^.

Initial crop budgets were determined using field trial agronomic data, and inputs and machinery price and cost data. All field operations from pre-seeding to harvest were included in the economic analysis. Input and output prices are summarized in Table 1. The net farm-gate price of $485 tonne^−1^ (t) for canola (i.e. the net cost of rail transportation and elevator handling) was used for canola gross income calculation^37^. The mean values for N and P (P_2_O_5_) were $1240 t^−1^ and $1200 t^−1^, respectively (Table 1).

We assumed that all farm machinery was operated by farmers and no custom work costs were included. Crop insurance, utilities and property taxes were not included in the total cost. For the effects of management zones and fertilizer treatments to be seen, participation in the Canada-provincial Crop Insurance Program was not included. No allowance was made for interest costs related to land equity. The skilled labour cost was assumed to be $25 h^−1^ for crop production. Farm practices followed by producers in western Canada, including equipment usage and field operation schedules, were established for each site. An average farm size of 907 ha was used to calculate work rates and farm machinery sizes^38^. For example, for fertilizer application, purchased price of machinery or equipment minus residual value was spread over optimal life of equipment and annual hours (h) of use (cost h^−1^) and investment and housing cost were added and then divided by work rate of machinery (hectares (ha) h^−1^) to calculate fixed cost for this operation per ha^38^. Fixed cost includes the cost of depreciation of the equipment due to use and years in service. It also includes an investment cost (e.g. the cost to borrow money to purchase the equipment and/or the lost interest revenue if that money had been invested), and housing/insurance costs^38^. Repair and maintenance, fuel and lubrication, and labour costs were also divided by work rate of the equipment to estimate each of these costs. Similar methods were used for each of the other field operations and added up to calculate field activity costs. Fertilizer costs, chemical costs, and seed costs were calculated by multiplying application rates and prices of each fertilizer, chemical, or seed. Combining and transportation costs from field to farm storage and from farm to first point of delivery were calculated in similar fashion assuming different truck sizes^38^. Grain truck capacity was assumed 9.5 t for field to yard and $9.5 t^−1^ for the first 5 km plus $0.92 t^−1^ for each additional kilometer (Table 1). Grain truck capacity was assumed 13 t for farm to first point of delivery with 100-km distance and 60 km h^−1^ speed. The grain truck was used 150 h per year. Transportation costs including fixed, repair and maintenance, fuel and lubrication, and labour costs based on crop yields were calculated similar to the other field operations. Costs and therefore NRs not only are affected by fertilizer treatments but also by the yield dependent costs as yield differences among zones or N treatments will affect transportation costs. Costs for auger and associated tractor use were also incorporated and it was assumed 20 min time was required to load and 5 min to unload a truck (Table 1). Interest rate on variable costs was assumed at 7%. This rate was used to reflect the range of public (5%) and private (10%) opportunity costs of capital. The analysis included the costs and returns for each site and year.

Yield data for 27 sites, from research conducted during 2014–2017 in Alberta, Saskatchewan and Manitoba, were related to replicate, yield zone, fertilizer treatment, and site-year in a linear model. Canola yield was analyzed for the period from 2014 to 2017 with a mixed model, where years, farms^39,40^, management zones and fertilizer treatments were defined as fixed effects and nested based on the experimental design, while experimental and observational error were expressed as random^40^. All interactions were specified in the model, with the exception of replicate. The model specified yield as the dependant continuous variable, with fertilizer treatment as ordinal, yield zone and site-year as nominal independent variables (fixed effects) and replicate as a nominal variable (random effect). Each field was considered as a site year in the linear model, which includes variability due to seasonal precipitation and temperature at that site. Canola yield was calculated from yield monitor data, with 4–7 subplots for each combination of replicate, yield zone, and fertilizer treatment in each field. Plot dimensions varied from field to field as influenced by seeder and swather width, and distribution of yield zones. Residuals were assessed for normality and homogeneity of variance, yield was log (base 10) transformed to meet these assumptions. Parameters for the linear model were calculated with maximum likelihood in a mixed model in statistical software JMP^35^. Denominator degrees of freedom for the linear model were calculated from subplots and independent variables with the Kenward–Roger first order approximation in JMP. Tukey’s HSD was used to compare means following regression analysis.

The economic analyses of site specific nitrogen management zones were based on both regression and statistical contrasts or analysis of variance (ANOVA). Estimated canola yield and NR were captured through change in nutrient, zones, and other variables such as farm management (site-year). Linear models of 2014–2016 data were specified and estimated as defined by Woolridge^41^. The regression model was specified as follows:

1 $$ y_{ft} = {\text{a }} + {\text{ bN }} + {\text{ cN}}^{{2}} + \, \lambda \times {\text{Zone }} + \, \theta \times {\text{Site}} - {\text{year,}} $$

where *y*_*ft*_ consists of crop yield or NR for field *f* over time *t*, N is nitrogen available (soil plus applied N), a is the constant coefficient, b and c are coefficients of N, zone represents spatial variability, and site-year represents site and temporal trend effects of each field over 2014–2016. λ and θ are coefficients of zones and site-year variables. Regression analysis was done by introducing categorical variables for zones or site-year and quantitative values for N rates. Modeled yield, crop prices and crop costs, and potential NR changes were then calculated for canola^42^. The Least Squares regression between yield or NR and zone as well as N rate and site-year was conducted using Eviews Econometric Software^43^ for each site-year or for all sites-years combined. The coefficients of the regression were tested and reported in Table 3.

Canola yield and NR were also analyzed with Proc Mixed^44,45^ for each site-year or combination of sites-years, and the error term was used in subsequent comparisons of means with the Tukey method in Proc PLM. The management zone and N rate, expressed as a percentage of recommended fertilizer rates for the target yield in the field, were fixed effects; replicate was random. Interactions with field, zone and N rate were not significant and not included in the final analyses in the results and discussion. Residuals from the model were reviewed to confirm normality and homogeneity of variance.

## Abbreviations

MZ: Management zones
N: Nitrogen
NR: Net revenue
NUE: Nitrogen use efficiency
GHG: Green house gasses

## References

[1] Rempel CB, Hutton SN, Jurke CJ (2014). Clubroot and the importance of canola in Canada. Can. J. Plant Pathol..

[2] LMC International. The economic impacts of canola on the Canadian economy. Report for: Canola council of Canada, Winnipeg, Canada. https://www.canolacouncil.org/media/584356/lmc_canola_10-year_impact_study_-_canada_final_dec_2016.pdf (2016).

[3] Canola Council of Canada. Nitrogen fertilizer management. http://www.canolacouncil.org/canola-encyclopedia/fertilizer-management/nitrogen-fertilizer-management/ (2017).

[4] Gan Y (2012). Carbon footprint of canola and mustard is a function of the rate of N fertilizer. Int. J. Life Cycle Ass..

[5] Pennock DJ, Walley FL, Solohub MP, Hnatowich G (2001). Topographically controlled yield response of canola to nitrogen fertilizer. Soil Sci. Soc. Am. J..

[6] Johnson CK, Mortensen DA, Wienhold BJ, Shanahan JF, Doran JW (2003). Site-specific management zones based on soil electrical conductivity in a semiarid cropping system. Agron. J..

[7] Zebarth BJ, Drury CF, Tremblay N, Cambouris AN (2009). Opportunities for improved fertilizer nitrogen management in production of arable crops in eastern Canada: A review. Can. J. Soil Sci..

[8] Mulla DJ (2013). Twenty five years of remote sensing in precision agriculture: Key advances and remaining knowledge gaps. Biosyst. Eng..

[9] Malhi SS, Johnson AM, Gill KS, Pennock DJ (2004). Landscape position effects on the recovery of 15N-labelled urea applied to wheat on two soils in Saskatchewan, Canada. Nutr. Cycling Agroecosyst..

[10] Mzuku M (2005). Spatial variability of measured soil properties across site-specific management zones. Soil Sci. Soc. Am. J..

[11] Holzapfel, C. B. Exploiting spatial and temporal variability in the Prairies. http://umanitoba.ca/faculties/afs/MAC_proceedings/proceedings/2009/Holzapfel_spatial_var_paper_2009.pdf (2009).

[12] Bramley RGV (2009). Lessons from nearly 20 years of Precision Agriculture research, development, and adoption as a guide to its appropriate application. Crop Pasture Sci..

[13] Fleming, K. L. & Westfall, D. G. Evaluating management zone technology and grid soil sampling for variable rate nitrogen application. In *Proceedings of the 5th International Conference on Precision Agriculture*, 179–184 (2000).

[14] Ge Y, Thomasson JA, Sui R (2011). Remote sensing of soil properties in precision agriculture: A review. Front. Earth Sci..

[15] Lowenberg-DeBoer J (2000). Economic Analysis of Precision Farming.

[16] Lambert, D. & Lowenberg-DeBoer, J. *Precision Agriculture Profitability Review*. (Purdue University, 2000).

[17] Manitoba Agricultural Services Corporation. MMPP—Variety yield data browser. https://www.masc.mb.ca/masc.nsf/mmpp_browser_variety.html (2018).

[18] Beckie HJ, Moulin AP, Pennock DJ (1997). Strategies for variable rate nitrogen fertilization in hummocky terrain. Can. J. Soil Sci..

[19] Torpy, T. Production & economic benefits of variable rate nitrogen application in cropping systems. http://www.precisionagriculture.com.au/assets/Industry%20Project%20Final%20Report%20-%20Brendan%20Torpy.pdf (2011).

[20] Raun WR (2002). Improving nitrogen use efficiency in cereal grain production with optical sensing and variable rate application. Agron. J..

[21] Long DS, Engel RE, Carlson GR (2000). Method for precision nitrogen management in spring wheat: II. Implementation. Precis. Agric..

[22] Long DS, Whitmus JD, Engel RE, Brester GW (2015). Net returns from terrain-based variable-rate nitrogen management on dryland spring wheat in Northern Montana. Agron. J..

[23] Boyer CN, Brorsen BW, Solie JB, Raun WR (2011). Profitability of variable rate nitrogen application in wheat production. Precis. Agric..

[24] Mulla DJ, Bhatti AU, Hammond MW, Benson JA (1992). A comparison of winter wheat yield and quality under uniform versus spatially variable fertilizer management. Agric. Ecosyst. Environ..

[25] Walley F, Pennock D, Solohub M, Hnatowich G (2001). Spring wheat (*Triticum aestivum*) yield and grain protein responses to N fertilizer in topographically defined landscape positions. Can. J. Soil Sci..

[26] Babcock BA, Pautsch GR (1998). Moving from uniform to variable fertilizer rates on Iowa corn: Effects on rates and returns. J. Agric. Resour. Econ..

[27] Bongiovanni R, Lowenberg-DeBoer J (2004). Precision agriculture and sustainability. Precis. Agric..

[28] Tekin AB (2010). Variable rate fertilizer application in Turkish wheat agriculture: Economic assessment. Afr. J. Agric. Res..

[29] Hong N (2006). Remote sensing-informed variable-rate nitrogen management of wheat and corn: Agronomic and groundwater outcomes. Agron. J..

[30] Holzapfel CB (2009). Optical sensors have potential for determining nitrogen fertilizer topdressing requirements of canola in Saskatchewan. Can. J. Plant Sci..

[31] Diacono M, Rubino P, Montemurro F (2013). Precision nitrogen management of wheat: A review. Agron. Sustain. Dev..

[32] Li A (2016). A case study of environmental benefits of sensor-based nitrogen application in corn. J. Environ. Qual..

[33] Manitoba Agriculture. Nitrogen rate calculator for wheat, barley and canola. https://www.gov.mb.ca/agriculture/crops/soil-fertility/nitrogen-rate-calculator.html (2009).

[34] Ag Leader. *SMS Advanced*, version 17.2. Ames, Iowa (2017).

[35] Sall J, Stephens ML, Lehman A, Loring S (2017). JMP Start Statistics: A Guide to Statistics and Data Analysis using JMP.

[36] Zentner RP (2002). Economics of crop diversification and soil tillage opportunities in the Canadian prairies. Agron. J..

[37] Manitoba Agriculture. Guidelines for estimating crop production costs—2019. https://www.gov.mb.ca/agriculture/farm-management/production-economics/cost-of-production.html (2019).

[38] Saskatchewan Agriculture. 2018–2019 Farm Machinery Custom and Rental Rate Guide. Government of Saskatchewan. http://www.publications.gov.sk.ca/details.cfm?p=76527 (2018).

[39] Steel, R., Torrie, J., & Dickey, D. Principles and procedures of statistics: A biological approach. McGraw-Hill series in probability and statistics. 3rd ed. (1997).

[40] Schabenberger O, Pierce FJ (2001). Contemporary Statistical Models for the Plant and Soil Sciences.

[41] Woolridge JM (2002). Econometric Analysis of Cross-Sectional and Panel Data, Cambridge.

[42] Khakbazan M, Hamilton C, Elliott J, Yarotski J (2013). Economic analysis of agricultural nutrient management practices in the South Tobacco Creek Watershed in Manitoba, Canada. J. Soil Water Conserv..

[43] Eviews quantitative software. http://www.eviews.com/ (2018).

[44] SAS Institute Inc., S. I. SAS® 9.3 *Base SAS*. Second Edition ed. SAS Institute Inc., Cary, NC, USA (2014).

[45] SAS Institute Inc., S. I. *SAS/STAT® 13.2 User’s Guide*. SAS Institute Inc., SAS Institute Inc. JMP^®^ 14 Fitting Linear Models. Cary, NC: SAS Institute Inc. (2014).

